# Role of Sca2 and RickA in the Dissemination of Rickettsia parkeri in Amblyomma maculatum

**DOI:** 10.1128/IAI.00123-18

**Published:** 2018-05-22

**Authors:** Emma K. Harris, Krit Jirakanwisal, Victoria I. Verhoeve, Chanida Fongsaran, Chanakan Suwanbongkot, Matthew D. Welch, Kevin R. Macaluso

**Affiliations:** a Vector-Borne Disease Laboratories, Department of Pathobiological Sciences, School of Veterinary Medicine, Louisiana State University, Baton Rouge, Louisiana, USA; b Department of Molecular and Cell Biology, University of California, Berkeley, Berkeley, California, USA; Washington State University

**Keywords:** actin-based motility, *Amblyomma maculatum*, RickA, Rickettsia parkeri, Sca2

## Abstract

The Gram-negative obligate intracellular bacterium Rickettsia parkeri is an emerging tick-borne human pathogen. Recently, R. parkeri Sca2 and RickA have been implicated in adherence and actin-based motility in vertebrate host cell infection models; however, the rickettsia-derived factors essential to tick infection are unknown. Using R. parkeri mutants lacking functional Sca2 or RickA to compare actin polymerization, replication, and cell-to-cell spread *in vitro*, similar phenotypes in tick and mammalian cells were observed. Specifically, actin polymerization in cultured tick cells is controlled by the two separate proteins in a time-dependent manner. To assess the role of Sca2 and RickA in dissemination in the tick host, Rickettsia-free Amblyomma maculatum, the natural vector of R. parkeri, was exposed to wild-type, R. parkeri rickA::*tn*, or *R. parkeri sca2*::*tn* bacteria, and individual tick tissues, including salivary glands, midguts, ovaries, and hemolymph, were analyzed at 12 h and after continued bloodmeal acquisition for 3 or 7 days postexposure. Initially, ticks exposed to wild-type R. parkeri had the highest rickettsial load across all organs; however, rickettsial loads decreased and wild-type rickettsiae were cleared from the ovaries at 7 days postexposure. In contrast, ticks exposed to R. parkeri *rickA*::*tn* or R. parkeri *sca2*::*tn* had comparatively lower rickettsial loads, but bacteria persisted in all organs for 7 days. These data suggest that while RickA and Sca2 function in actin polymerization in tick cells, the absence of these proteins did not change dissemination patterns within the tick vector.

## INTRODUCTION

Members of the spotted fever group (SFG) Rickettsia are obligate intracellular bacteria transmitted by ticks vertically (between life cycle stages) and horizontally (between ticks) via a vertebrate host. In horizontal acquisition, ticks imbibe an infectious bloodmeal from the vertebrate host, allowing the rickettsiae to enter the gut and then, through undefined mechanisms, disseminate throughout the tick to infect organs central to transmission, including the ovaries (vertical) and salivary glands (horizontal). The ability of individual Rickettsia species to successfully infect and be transmitted by a tick host varies by both Rickettsia and tick species (1). Transmission of SFG Rickettsia to a vertebrate host during tick feeding can result in disease ranging from a mild, self-limiting infection to death (2, 3). The incidence of tick-borne SFG rickettsiosis is on the rise due to increased recognition among physicians, increased geographic distribution of tick vectors, and the emergence of rickettsial pathogens (2, 4, 5). Among the more recently recognized pathogens is Rickettsia parkeri, with at least 35 confirmed human cases, not including patients likely misdiagnosed with classic Rocky Mountain spotted fever (6). The molecular factors contributing to rickettsial colonization, maintenance, and transmission within tick vectors have not been identified.

It has recently been demonstrated that disruption of two key proteins leading to R. parkeri actin-based motility (ABM) negatively impacts intracellular bacterial movement and therefore dissemination from cell to cell in *in vitro* models of mammalian infection (7). One of these proteins, RickA, is a nucleation promoting factor that functions by activating the host cell Arp2/3 complex to mediate actin branching and ABM (7, 8). A second protein, surface cell antigen 2 (Sca2), has also been shown to act as a formin-like mediator of ABM and contributes to mammalian cell adhesion (9–11). Utilizing transposon mutagenesis to generate two strains of R. parkeri, one lacking expression of full-length Sca2 and the other lacking RickA, the current model of R. parkeri actin-based motility suggests that RickA coordinates early-phase motility (15 to 30 min postinfection), giving rise to short actin tails and slow bacterial movement. Alternatively, late-phase motility (24 to 48 h postinfection) is mediated by Sca2, resulting in more elongated actin tails and increased rickettsial velocity within the cell (7). While progress has been made toward understanding the role of rickettsial proteins in vertebrate host cell infection, their function in arthropod cells and during infection and dissemination in the tick vector is unknown.

In this study, the phenotypes of RickA- and Sca2-deficient R. parkeri were assessed in an arthropod host cell background *in vitro* to determine if strategies of ABM utilized in the tick host are similar to those reported for vertebrate host cells. Additionally, infection and dissemination dynamics of R. parkeri wild-type, *rickA*::*tn*, and *sca2*::*tn* strains in the tick vector Amblyomma maculatum were evaluated to determine if ABM orchestrated by rickettsial Sca2 and RickA contributes to R. parkeri dissemination within its tick host. Similar phenotypes were observed by comparing vertebrate and tick host cell backgrounds, and while all strains were able to disseminate in the tick after acquisition, the wild-type strain resulted in a greater bacterial load with a diminished ability to persist in tick reproductive tissue.

## RESULTS

### Actin polymerization of R. parkeri in arthropod cells is comparable to that in mammalian cells.

**FIG 1:**
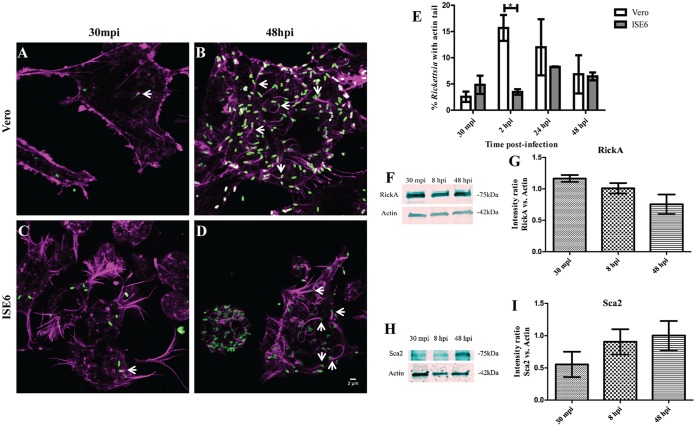
Actin polymerization of R. parkeri in Vero and ISE6 cells and expression of Sca2 and RickA in ISE6 cells. (A and B) Wild-type R. parkeri (green) polymerizing actin (magenta) in Vero cells at 30 mpi and 48 hpi. (C and D) Wild-type R. parkeri (green) polymerizing actin (magenta) in ISE6 cells 30 mpi and 48 hpi. White scale bar, 2 μm. Arrows indicate Rickettsia polymerizing actin. (E) Percentage of wild-type R. parkeri present in Vero and ISE6 cells with an actin tail at 30 mpi and 2, 24, and 48 hpi. Bacteria with and without actin tails were recorded at each time point postinfection in order to determine the profile of R. parkeri actin polymerization. Error bars represent the standard errors of the means. Data are representative of two replicates per experiment and two independent experiments. Ten images taken across all experimental replicates were used in analyses. (F and G) Western blot (F) and corresponding graph (G) of RickA expression normalized against ISE6 expression of β-actin at 30 mpi and 8 and 48 hpi for wild-type R. parkeri. (H and I) Western blot (H) and corresponding graph (I) of Sca2 expression normalized against ISE6 expression of β-actin at 30 mpi and 8 and 48 hpi for wild-type R. parkeri. Statistical analysis consisted of a one-way ANOVA followed by Tukey's *post hoc* analysis (G and I) or an unpaired *t* test (E), with a *P* value of <0.05 considered significant (denoted by an asterisk). Data are representative of two replicates per experiment and two independent experiments.

To define the temporal pattern of R. parkeri motility, ISE6 cells were infected and ABM assessed at several time points. Tandem experiments in Vero cells were completed to act as a positive control for previously established actin polymerization patterns (7). Rickettsia was observed to actively polymerize actin at both 30 min postinfection (mpi) and 48 h postinfection (hpi) in Vero and ISE6 cells (Fig. 1A to D). Less than 5% of wild-type R. parkeri was observed to polymerize actin after 30 min of infection in ISE6 cells (Fig. 1E). Maximum polymerization was observed at 2 hpi in Vero cells and at 24 hpi in ISE6 cells (Fig. 1E). High-magnification images of ABM in ISE6 cells were visualized at 48 hpi, demonstrating a similarity to that previously shown in mammalian cells (see Fig. S1 in the supplemental material) (7–9, 12, 13). Expression of RickA and Sca2 in wild-type R. parkeri in tick cells matched observations in Vero cells with nonsignificant inverse expression of RickA and Sca2 (Fig. 1F to I). Overall, these data show that wild-type R. parkeri actin polymerization occurs in both Vero and ISE6 cells.

### Rickettsial mutants lacking Sca2 or RickA replicate similarly to wild-type R. parkeri in tick cells.

**FIG 2:**
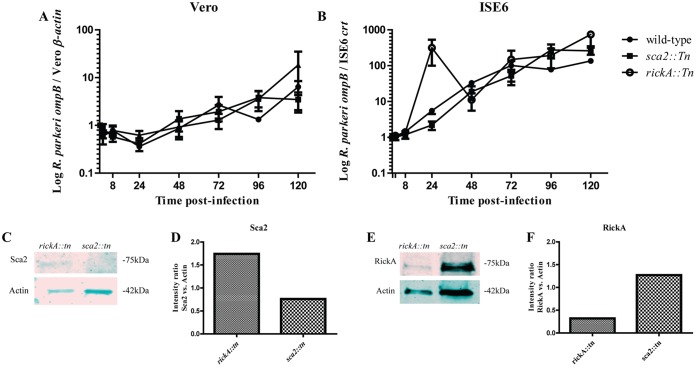
Growth curves for R. parkeri wild-type, *sca2*::*tn*, and *rickA*::*tn* strains in Vero (A) and ISE6 (B) cells. Data are fold change compared to initial input. Collection times consisted of 30 mpi and 2, 8, 24, 48, 72, 96, and 120 hpi. There was a lack of statistical significance at all time points for *R. parkeri sca2*::*tn* and *rickA*::*tn* strains compared to wild-type R. parkeri across both cell types. Data are representative of three replicates per experiment and two independent experiments. A Kruskal-Wallis test with Dunn's *post hoc* analysis was completed in GraphPad Prism software. A *P* value of <0.05 was considered significant. Western blots showing expression or lack of expression of Sca2 (C) and RickA (E) in *R. parkeri sca2*::*tn* and rickA::*tn*. Densitometric analysis of Sca2 (D) and RickA (F) expression in respective mutant strains was completed by normalizing expression of each protein against expression of β-actin in Vero cells.

To assess potential differences in growth kinetics between wild-type R. parkeri and *sca2*::*tn* or *rickA*::*tn* strains in tick-derived cells, rickettsiae were grown in Vero and tick cells for 120 h. Replication of both *R. parkeri sca2*::*tn* and R. parkeri rickA::*tn* strains was similar to that of the wild-type R. parkeri in Vero and ISE6 cell lines (Fig. 2A and B). Overall replication rates were greater in ISE6 cells than in Vero cells, with rickettsial density increasing approximately 100-fold for all three strains. However, there was no significant difference between the replication of the mutant strains and wild-type R. parkeri in either host cell background. Therefore, rickettsial Sca2 and RickA are not required for replication within cells *in vitro*, independent of host cell origin. Additionally, assessment of expression of Sca2 and RickA expression in respective mutant strains was carried out via Western blotting. Results showed that Sca2 expression was not present in *R. parkeri sca2*::*tn* samples but was consistently observed at 75 kDa in R. parkeri rickA::*tn* samples (Fig. 2C). Conversely, RickA expression was detected in *R. parkeri sca2*::*tn* samples and weakly in R. parkeri rickA::*tn* samples (Fig. 2D).

### Time-dependent ABM occurs in tick cells *in vitro*.

**FIG 3:**
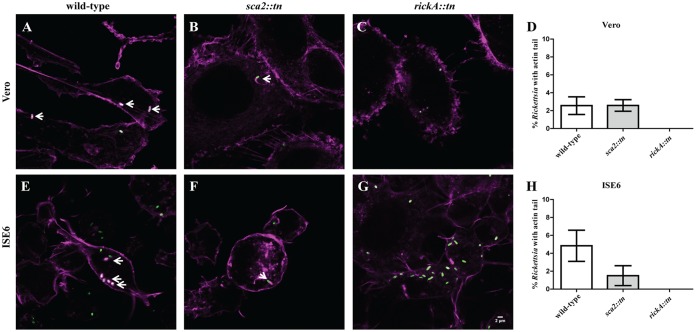
Actin polymerization profile of wild-type R. parkeri compared to *R. parkeri sca2*::*tn* and rickA::*tn* in Vero and ISE6 cells at 30 mpi. Rickettsia (green) actively polymerizing actin (magenta) in Vero (A to C) and ISE6 (E to G) cells is shown. This assay was repeated for R. parkeri wild-type (A and E), *R. parkeri sca2*::*tn* (B and F), and R. parkeri rickA::*tn* (C and G) strains. (D and H) Graphical representation of percent Rickettsia with an actin tail in Vero (D) and ISE6 (H) cells. Data are representative of two replicates per experiment and two independent experiments. Statistical analysis consisted of a *t* test. *P* < 0.05. White scale bar, 2 μm. Arrows indicate Rickettsia polymerizing actin.

**FIG 4:**
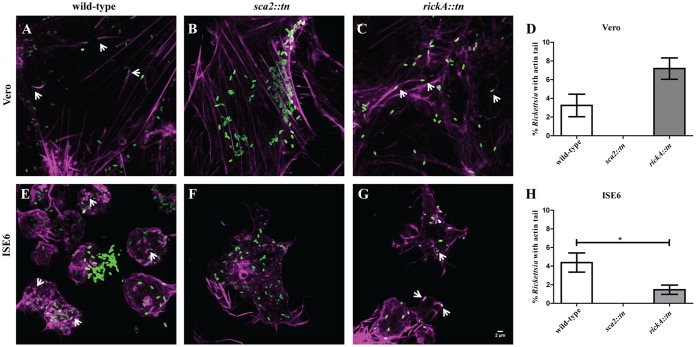
Actin polymerization of wild-type R. parkeri compared to that of *R. parkeri sca2*::*tn* and rickA::*tn* in Vero and ISE6 cells at 48 hpi. Rickettsia (green) actively polymerizing actin (magenta) in Vero (A to C) and ISE6 (E to G) cells is shown. Wild-type R. parkeri (A and E), *R. parkeri sca2*::*tn* (B and F), and R. parkeri rickA::*tn* (C and G) strains are depicted. (D and H) Graphical representation of percent Rickettsia with actin tails in Vero (D) and ISE6 (H) cells. Data are representative of two replicates per experiment and two independent experiments. Statistical analysis consisted of a *t* test, with a *P* value of <0.05 being significant. White scale bar, 2 μm. Arrows indicate Rickettsia polymerizing actin.

Previous analyses in mammalian cells suggest that early-phase (30 mpi) ABM is driven by RickA and late-phase ABM (24 to 48 hpi) is initiated by Sca2 (7). Wild-type R. parkeri demonstrated actin polymerization at 30 mpi in both cell types (Fig. 3A and E). Utilizing fluorescence microscopy to visualize infected cells, it was determined that *R. parkeri sca2*::*tn* also employs actin polymerization at 30 mpi in both Vero and ISE6 cells (Fig. 3B and F). However, for R. parkeri rickA::*tn*, ABM was not observed at 30 mpi (Fig. 3C and G), demonstrating that similar to mammalian *in vitro* infection, early-phase rickettsial motility in arthropod cells is coordinated by RickA. The percentage of total rickettsiae polymerizing actin in either cell type did not differ between wild-type and mutant R. parkeri at 30 mpi (Fig. 3D and H). At 48 hpi, actin polymerization was observed in Vero and ISE6 cells for both wild-type R. parkeri and rickA::*tn* strains (Fig. 4A, C, E, and F), while the *sca2*::*tn* strain did not polymerize actin (Fig. 4B and F). In addition to no actin polymerization by *R. parkeri sca2*::*tn*, there was a significant decrease in ABM observed in the *rickA*::*tn* strain compared to that of wild-type R. parkeri in tick cells only (Fig. 4D and H). Similar to the phenotype observed in Vero cells, actin polymerization in tick cells is controlled by two separate proteins in a time-dependent manner.

### Cell-to-cell spread of R. parkeri rickA::*tn* is significantly reduced in tick cells.

**FIG 5:**
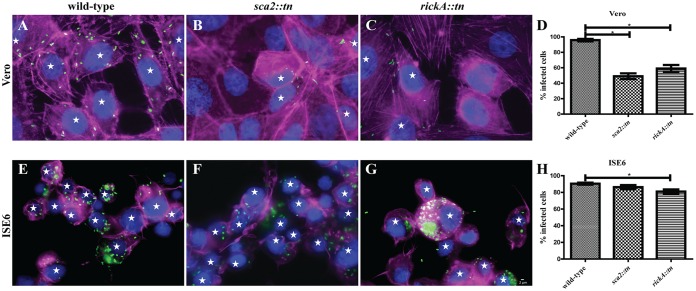
Percentage of R. parkeri-infected Vero and ISE6 cells at 24 hpi. Epifluorescence microscopy of Vero (A to C) or ISE6 (E to G) cells infected with R. parkeri wild type (A and E), *R. parkeri sca2*::*tn* (B and F), and R. parkeri rickA::*tn* (C and G). Cells were stained for Rickettsia (green), nuclear material (blue), and actin (magenta). (D and H) Graphical representation of percent infected Vero (D) and ISE6 (H) cells. Data are representative of two replicates per experiment and two independent experiments. Statistical analysis consisted of a one-way ANOVA with Tukey's *post hoc* analysis. *P* < 0.05. White scale bar, 2 μm. Stars indicate cells infected with Rickettsia.

A cell-to-cell spread assay was employed to determine the effect that loss of RickA or Sca2 had on dissemination of R. parkeri in cultured cells. A significant decrease in rickettsial spread to neighboring cells was observed for both R. parkeri rickA::*tn* and *sca2*::*tn* in Vero cells (Fig. 5A to D). In ISE6 cells, a significant decrease in rickettsial dissemination was observed only for R. parkeri rickA::*tn* (Fig. 5E to H). This demonstrates that rickettsial infection in mammalian, but not arthropod, cells is impacted by the disruption of both *rickA* and *sca2*.

### Dissemination of R. parkeri in A. maculatum does not depend on Sca2 or RickA.

**FIG 6:**
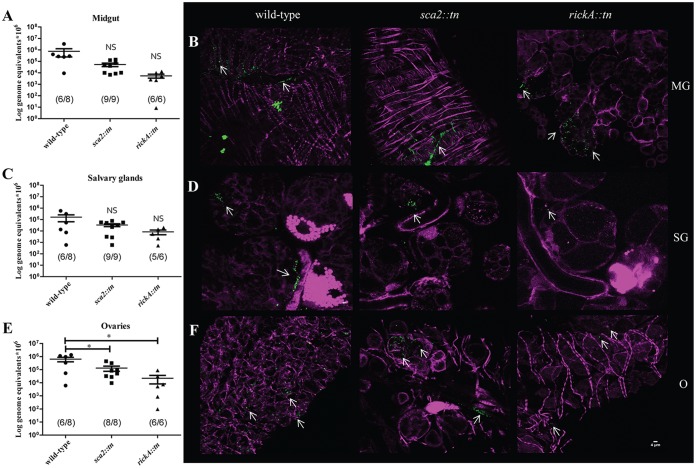
Dissemination of R. parkeri wild-type, *sca2*::*tn*, and rickA::*tn* in A. maculatum at 12 hpe. Mean rickettsial load as quantified by qPCR for the midgut (A), salivary glands (C), and ovaries (E). Inset values represent the number of infected ticks over the number of total ticks tested via qPCR. (B) Confocal microscopy of midgut (B, top), salivary glands (D, middle), and ovaries (F, bottom), corresponding to the data presented in panel A for the R. parkeri wild-type (left), *sca2*::*tn* (center), and *rickA*::*tn* (right) strains. All tissues were stained for Rickettsia (green) and actin (magenta). Statistical analysis consisted of a one-way ANOVA followed by Tukey's *post hoc* analysis, with a *P* value of <0.05 being considered significant (denoted by an asterisk). Error bars represent the SEM. Means are represented by the bar between the SEM. NS indicates nonsignificant data sets compared to wild-type data. Data and images are representative of 2 independent experiments. White scale bar, 4 μm. Arrows indicate rickettsiae.

The role of RickA and Sca2 was investigated *in vivo* by infecting A. maculatum ticks via a previously published capillary feeding technique to expose ticks to a dose of 5 × 10^7^ rickettsiae/μl (1, 14). At 12 h postexposure (hpe), among the treatment groups, rickettsiae were detected in 75 to 100% of exposed ticks, demonstrating effective acquisition of rickettsiae. There was no significant difference in rickettsial density between organs within each strain (i.e., levels of wild-type R. parkeri in the midgut versus that in the salivary glands). In the midgut, no significant difference between wild-type R. parkeri load and *R. parkeri sca2*::*tn* or rickA::*tn* was detected. In the ovaries, wild-type R. parkeri had a significantly higher rickettsial burden than either of the mutant strains (Fig. 6A, C, and E). Microscopic evaluation of each organ (Fig. S2) for the presence of rickettsiae and actin tail formation was conducted; however, while all three strains of Rickettsia were detected, no evidence of ABM was observed (Fig. 6B, D, and F and Fig. S3). These data demonstrate that rickettsial dissemination into all major organs occurs after 12 h of capillary feeding. Additionally, rickettsial mutants are not deficient in infecting the midgut or salivary glands compared to the wild-type parent strain. However, infection in the ovaries was significantly lower at 12 h than that of the wild-type strain. While differences in bacterial density were observed, the lack of a distinct dissemination phenotype suggests that neither Sca2 nor RickA alone is essential for early infection in the tick host.

**FIG 7:**
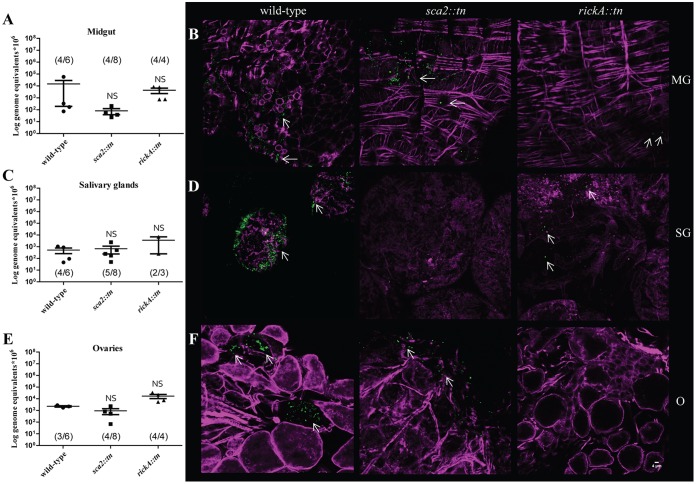
Dissemination of R. parkeri wild type, *sca2*::*tn*, and rickA::*tn* at 3 dpe. (A, C, and E) Mean rickettsial load as quantified by qPCR for the midgut (A), salivary glands (C), and ovaries (E). Inset values represent the number of infected ticks over the number of total ticks tested via qPCR. (B) Confocal microscopy of midgut (B, top), salivary glands (D, middle), and ovaries (F, bottom), corresponding to the data presented in panel A for R. parkeri wild-type (left), *sca2*::*tn* (center), and *rickA*::*tn* (right) strains. All tissues were stained for Rickettsia (green) and actin (magenta). Statistical analysis consisted of a one-way ANOVA followed by Tukey's *post hoc* analysis, with a *P* value of <0.05 considered significant (denoted by an asterisk). Error bars represent the SEM. The mean is represented by the bar between the SEM. NS indicates nonsignificant data sets compared to wild-type data. Data and images are representative of 2 independent experiments, excepting the R. parkeri rickA::*tn* strain. White scale bar, 4 μm. Arrows indicate rickettsiae.

Following the establishment of a dissemination profile at 12 hpe, an assessment of the contributing role of Sca2 and RickA in bacterial persistence within the feeding tick was investigated. After 3 days of additional bloodmeal acquisition, ticks (*n* = 3 to 8 ticks/strain) were forcibly removed from the host and rickettsial infection was assessed. For this group, 50 to 100% of the individual organs exposed to either wild-type R. parkeri or mutant strains were infected. Compared to results at 12 hpe, rickettsial density in each organ was 1 to 2 logs lower at 3 days postexposure (dpe). Midguts, salivary glands, and ovaries had variable densities of all strains of R. parkeri, with no significant differences between each organ or rickettsial strain (Fig. 7A, C, and E). These data demonstrate that disseminated R. parkeri infection is sustained 3 dpe in a feeding tick, which was also confirmed via microscopy (Fig. 7B, D, and F). Numbers of R. parkeri rickA::*tn*-infected ticks were lower at 3 dpe due to death postexposure; however, no distinct phenotype was observed for rickettsial mutants compared to wild-type R. parkeri, further indicating dissemination in the vector occurs independently of RickA or Sca2.

**FIG 8:**
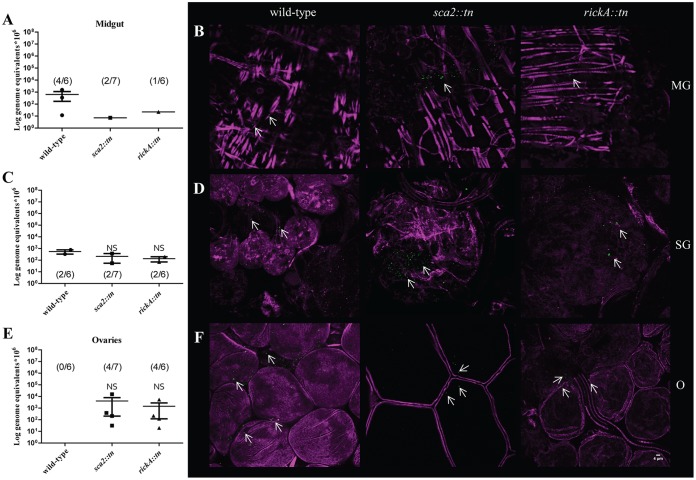
Dissemination of R. parkeri wild type, *sca2*::*tn*, and rickA::*tn* at 7 dpe. (A, C, and E) Mean rickettsial load as quantified by qPCR for the midgut (A), salivary glands (C), and ovaries (E). Inset values represent the number of infected ticks over the number of total ticks tested via qPCR. (B) Confocal microscopy of midgut (B, top), salivary glands (D, middle), and ovaries (F, bottom), corresponding to the data presented in panel A for R. parkeri wild-type (left), *sca2*::*tn* (center), and *rickA*::*tn* (right) strains. All tissues were stained for Rickettsia (green) and actin (magenta). Statistical analysis consisted of a one-way ANOVA followed by Tukey's *post hoc* analysis, with a *P* value of <0.05 considered significant (denoted by an asterisk). Error bars represent the SEM. The mean is represented by the bar between the SEM. NS indicates nonsignificant data sets compared to wild-type data. Data and images are representative of 2 independent experiments. White scale bar, 4 μm. Arrows indicate rickettsiae.

Female A. maculatum can feed for an extended period of time (∼10 days); therefore, to further investigate the dissemination and persistence pattern of wild-type R. parkeri compared to *R. parkeri sca2*::*tn* and *rickA*::*tn*, ticks (*n* = 6 to 7 ticks/strain) were allowed to feed on host for 7 dpe. Infection of A. maculatum organs at 7 dpe ranged from 0 to 67% of exposed tick tissues. Rickettsial strains were 1 to 2 logs lower at 7 dpe than at 3 dpe for each organ. All strains of R. parkeri were detected by qPCR in the midgut and salivary glands for 7 dpe (Fig. 8A and C), demonstrating a persistent infection phenotype in these organs independent of infecting strain. In tick ovaries, *R. parkeri sca2*::*tn* and *rickA*::*tn*, but not wild type, were detected by qPCR (Fig. 8E). Rickettsiae were identified by microscopy in all tissues (Fig. 8B, D, and F), even in the absence of qPCR-positive samples for wild-type R. parkeri; however, staining was not appreciable when compared to no-primary-antibody control images for each set of organs, which identified limited, nonspecific binding (Fig. S4). Statistical inferences could not be made comparing the wild-type R. parkeri and the *R. parkeri sca2*::*tn* and R. parkeri rickA::*tn* strains due to low numbers of infected ticks at 7 dpe. Although the mean rickettsial load decreased during tick feeding postexposure, R. parkeri organisms lacking expression of Sca2 and RickA were still able to infect and persist in A. maculatum.

**FIG 9:**
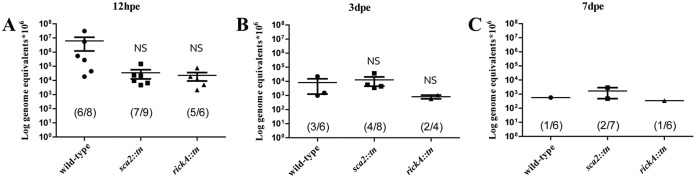
Presence of R. parkeri wild type, *sca2*::*tn*, and rickA::*tn* in the hemolymph of exposed A. maculatum. Mean rickettsial load was quantified at 12 h (left), 3 days (middle), and 7 days (right) postexposure. Statistical analysis consisted of a one-way ANOVA followed by Tukey's *post hoc* analysis, with a *P* value of <0.05 considered significant (denoted by an asterisk). Error bars represent the SEM. The mean is represented by the bar between the SEM. NS indicates nonsignificant data sets compared to wild-type data. Data are representative of two replicates per experiment and two independent experiments.

Evidence of infection in the hemolymph suggests that, much like initial bacterial numbers in the other tick organs assessed here, rickettsial load was highest at 12 hpe (Fig. 9A). This density decreased approximately 3 logs for ticks infected with wild-type R. parkeri by 3 dpe (Fig. 9B). At 7 dpe, mean rickettsial load and total number of ticks with detectable wild-type R. parkeri were further decreased (Fig. 9B). Overall, there was a 7 log decrease in rickettsial load in the hemolymph from 12 hpe to 7 dpe for ticks infected with wild-type R. parkeri. For A. maculatum infected with R. parkeri rickA::*tn*, rickettsial infection decreased only by 1 log from 12 hpe to 3 dpe (Fig. 9A and B). At 7 dpe, although rickettsial load did not significantly decrease, only one tick retained infection (Fig. 9C). Similarly, there was an approximately 1 log decrease in rickettsial load in ticks infected with R. parkeri *sca2*::*tn* between 12 hpe and 3 dpe, with 50% (4/8) of the ticks with detectable rickettsiae at the latter time point (Fig. 9A and B). At 7 dpe, only 29% (2/7) of the ticks exposed to the R. parkeri *sca2*::*tn* strain remained infected, with overall rickettsial load decreasing approximately 1.5 logs compared to 12 hpe. The incidence and rickettsial load decreased in tick hemolymph throughout blood feeding after exposure to rickettsiae.

## DISCUSSION

As intracellular bacteria, SFG Rickettsia require host cells to replicate. The critical initial steps in rickettsial pathogenesis include bacterial recognition of, and attachment to, target cells. In vertebrates, SFG Rickettsia infect host endothelium, as well as a wide spectrum of phagocytic and nonphagocytic cells (15). Subsequent to invasion of the host cell, rickettsial pathogens undergo ABM to promote cell-to-cell spread during infection *in vitro* (11). Unique to the SFG Rickettsia is the presence of two actin-polymerizing proteins. RickA, an activator of the host Arp2/3 complex, was initially proposed to drive motility (8, 16). In addition to being implicated in adherence and invasion, Sca2 was also shown to play a key role in motility, and both motility pathways facilitate cell-to-cell spread in vertebrate cell lines (7, 17). In the current study, a series of experiments was designed to determine if R. parkeri Sca2 and RickA display comparable phenotypes in arthropod and mammalian cell lines. *In vitro*, wild-type R. parkeri infection in ISE6 cells resulted in larger amounts of initial expression of RickA, which decreased over time. Conversely, expression of Sca2 was low at initial infection time points and increased with time. These observations in tick cells, along with production of actin tails, were consistent with previous studies using only mammalian cells (7). Independent of RickA or Sca2 expression, wild-type and mutant strains shared similar growth kinetics among strains but not between cell lines. In the tick-derived Ixodes scapularis cells, replication rates were greater than those in Vero cells. Reasons for the marked difference are not clear but may be due to variations in cell culture conditions, which can influence rickettsial growth or the potential for physiological coupling between SFG Rickettsia and ticks (18, 19).

Further assessment of actin polymerization by R. parkeri strains lacking functional RickA or Sca2 revealed that infection with R. parkeri rickA::*tn* resulted in a loss of actin-based polymerization in both ISE6 and Vero cells at 30 mpi; however, actin-based polymerization was observed at 48 hpi. Alternatively, cells infected with *R. parkeri sca2*::*tn* displayed early actin-based polymerization but no late-phase actin polymerization in both ISE6 and Vero cells. Together, the data suggest that R. parkeri strains deficient in functional RickA or Sca2 display conserved phenotypes in arthropod and mammalian cells *in vitro*. Observed deficiencies in ABM were expected, as the host protein complexes utilized for actin-based motility, e.g., Arp2/3 complex, are functional in tick cells (20, 21). Interestingly, compared to wild-type R. parkeri, decreased late-phase actin polymerization was observed for R. parkeri rickA::*tn*, coinciding with reduced cell-to-cell spread. The interaction of specific rickettsial proteins with distinct cell types is not well defined, and while the utility of using rickettsial transformants to examine their interaction with tick cells is promising, it is important to consider that tick-derived cell lines typically originate from embryonic tick cells and often contain multiple, undefined cell types (22, 23). Thus, to expand on the *in vitro* observations, an *in vivo* infection bioassay was employed in order to determine the contribution of RickA and Sca2 to infection and persistence within the tick host.

The process of infection of ticks by SFG Rickettsia has been visualized using genetically modified rickettsiae. The SFG endosymbiont Rickettsia monacensis was transformed to express green fluorescent protein (GFP) to observe rickettsial infection in unfixed tick tissues after introduction by capillary feeding (24). While rickettsial survival and dissemination were tick species dependent, GFP-expressing organisms were detected in the gut of ticks up to 4 weeks postexposure (24). In the current study, an established capillary feeding technique (14) was utilized to expose A. maculatum to R. parkeri wild-type, *rickA*::*tn*, or *sca2*::*tn* strains and produce an infection which was temporally monitored within individual tick tissues. At 12 hpe, R. parkeri wild-type, *rickA*::*tn*, and *sca2*::*tn* strains were detected in all organs. Significantly higher levels of wild-type R. parkeri than of either mutant strain were present in the ovaries, suggesting that at initial stages of infection RickA and Sca2 are important in rapid dissemination. As the capillary feeding bioassay is beneficial in providing a natural route of exposure (i.e., during bloodmeal acquisition), determining the exact amount of rickettsiae each tick initially receives is not possible and may account for the variation in rickettsial loads in individual organs over time. The nonsignificant variation in rickettsial load in the midgut at 12 hpe suggests that ticks received equivalent amounts of rickettsiae, and the relatively rapid dissemination suggests that the process occurs independently of actual exposure dose. Dissemination to all organs at 12 hpe was a novel observation and highlights the complex nature of rickettsial acquisition by the feeding tick, which consists of multiple organ structures, including the tracheal system, which has previously been hypothesized as a route for producing disseminated infection (24). Rickettsia-exposed ticks were allowed to resume bloodmeal acquisition, and at 3 dpe, ticks had comparable levels of rickettsiae present in gut, salivary glands, and ovaries, suggesting the lack of functional RickA or Sca2 did not generate a distinct phenotype. However, at 7 dpe, rickettsial load and the overall percentage of infected ticks were lower than those at 12 hpe and 3 dpe, suggesting that persistent infection waned with prolonged tick feeding. Among the strains, during the exposure period wild-type R. parkeri retained the highest infection load in the midgut and the salivary glands but was cleared from the ovaries. Whereas rickettsial loads for both *R. parkeri sca2*::*tn* and rickA::*tn* were diminished in all tick organs assessed, they were detectable 1 week postexposure. Thus, these results suggest that the loss of RickA or Sca2 does not negatively impact, and may favor, the persistence phenotype of R. parkeri in the tick host.

Both arthropod and rickettsial factors likely contribute to rickettsial dissemination and persistence in the tick host. Upon feeding and rickettsial challenge, upregulation of stress and immune responses are observed in ticks in a tissue-specific manner (25–27). Tick immune molecules may have led to ovarian clearance of wild-type R. parkeri, which displays no defect in motility compared to R. parkeri *sca2*::*tn* and *R. parkeri rickA*::*tn*, suggesting a role for these proteins in tick immune response induction. A similar hypothesis has been proposed for Rickettsia peacockii, a species that lacks expression of RickA that was originally thought to confer a persistence phenotype in the ovaries of its tick host, Dermacentor andersoni (28). However, genome sequencing has also shown that R. peacockii possesses deletions in genes leading to the disruption of *ompA* and *Sca1*, among other genes (29). Thus, the reason R. peacockii persists in tick ovaries is unknown at the molecular level, but the ABM-deficient R. parkeri mutants utilized in this study support the concept that decreased motility facilitates stable vertical transmission through rickettsial persistence in tick reproductive tissues due to limited exposure to tick immune factors.

Previous data have shown that actin polymerization is the primary mode of rickettsial movement intracellularly (12, 30, 31). The *in vitro* data put forth demonstrate that, like mammalian models, Sca2 and RickA contribute to a pattern of rickettsial ABM in tick cells. However, no implications for ABM by Rickettsia in tick hosts could be discerned in the present study. Furthermore, less than 20% of wild-type rickettsiae were observed to polymerize actin at any one time postinfection; thus, it is possible that the microscopy employed failed to accurately capture rickettsial ABM events. Alternatively, it is possible that additional proteins aid in rickettsial dissemination and, therefore, persistence. For instance, Sca4 has recently been identified as a secreted factor that leads to rickettsial protrusion and infection of neighboring cells independent of ABM (32). The role of Sca4 in tick infection and dissemination requires further examination.

In summary, using an appropriate tick host/SFG Rickettsia system, the data suggest that while Sca2 and RickA function similarly in arthropod and mammalian cell lines, they are not essential for initial infection and dissemination of Rickettsia into tick organs and may influence persistence in tick ovaries. These studies can serve as a model for assessing rickettsial factors that have the potential to contribute to the infection and transmission dynamics of SFG Rickettsia within an actively feeding tick host. Furthermore, the roles of Sca2 and RickA, and other suspected SFG Rickettsia virulence determinants, such as OmpA (33), as transmission factors can be assessed by examining the interface between ticks and vertebrate hosts.

## MATERIALS AND METHODS

### Rickettsial strains and purification.

Rickettsia parkeri wild-type (strain Portsmouth), *R. parkeri sca2*::*tn*, and R. parkeri rickA::*tn* strains were derived and propagated as previously published (7, 34, 35). For *in vitro* and *in vivo* bioassays, low-passage-number (≤5) Rickettsia organisms were semipurified as previously published (18). Briefly, infected cells were lysed using a 27-gauge needle, followed by centrifugation to separate Rickettsia from cell debris, and, finally, supernatant was passed through a 2-μm syringe filter (Whatman). Rickettsial enumeration was performed using a BacLight Live/Dead viability kit (Molecular Probes) with a Petroff-Hausser counting chamber (Hausser Scientific Company) and viewed on a fluorescence microscope (Leica) (36).

### *In vitro* infection assays.

Vero cells and Ixodes scapularis-derived embryonic (ISE6) cells were cultured as previously described (35, 37). For *in vitro* examination of rickettsial ABM and cell-to-cell spread associated with R. parkeri wild-type, *sca2*::*tn*, and *rickA*::*tn* strains via microscopy, cells were seeded onto glass coverslips in 24-well plates. Vero cells were seeded at a density of 5 × 10^4^ cells per well and incubated at 32°C for 24 h prior to infection. Additionally, ISE6 cells were seeded at 2 × 10^5^ cells per well and incubated at 32°C prior to infection. Cells were infected at a multiplicity of infection (MOI) of 50. Contact between the bacteria and host cell was induced by centrifugation at 500 × *g* for 5 min at room temperature. One hour postinoculation (hpi), medium was replaced to remove unbound bacteria. Coverslips containing infected cells were collected at 30 min postinfection (mpi) and 2, 24, and 48 hpi. All coverslips were rinsed once with phosphate-buffered saline (PBS), followed by immunofluorescence staining.

For growth curve analysis of R. parkeri wild type, *sca2*::*tn*, and *rickA*::*tn*, Vero and ISE6 cells were seeded in 96-well plates. Vero cells were seeded at a density of 1 × 10^3^ cells per well and incubated at 34°C prior to experimentation. ISE6 cells were seeded at a density of 5 × 10^4^ cells per well and incubated at 32°C prior to experimentation. All cells were infected with rickettsiae at an MOI of 50 and centrifuged at 500 × *g* for 5 min at room temperature to induce bacterium-host cell contact. At 1 hpi, medium was replaced to remove unbound bacteria. Cells were collected at 30 mpi and 2, 8, 24, 48, 72, 96, and 120 hpi. Vero cells were dislodged by washing cells once with PBS, incubating with trypsin-EDTA, and adding fresh media to collect cells. ISE6 cells were recovered by dislodging cells with PBS. All infected cell samples were centrifuged at 2,100 × *g* for 10 min at 4°C, the supernatant removed, and pellets stored at −20°C until processed for genomic DNA (gDNA) extraction.

For protein analysis of Sca2 and RickA expression in wild-type R. parkeri via immunoblotting, Vero and ISE6 cells were seeded in 6-well plates. Vero cells were seeded at a density of 1 × 10^5^ cells per well and incubated at 34°C prior to experimentation. ISE6 cells were seeded at a density of 5 × 10^6^ cells per well and incubated at 32°C prior to experimentation. All cells were infected with rickettsiae at an MOI of 50 and centrifuged at 500 × *g* for 5 min at room temperature to induce bacterium-host cell contact. At 1 hpi, medium was replaced to remove unbound bacteria. Cells were collected at 30 mpi and 8 and 48 hpi. Infected cells were collected by replacing media with PBS, lifted with a cell scraper, washed twice in PBS via centrifugation at 2,100 × *g* for 5 min at 4°C, and resuspended in radioimmunoprecipitation assay (RIPA) buffer containing protease inhibitor cocktail (Roche). Samples were sonicated, followed by low-speed centrifugation to remove cell debris, and stored at −80°C prior to SDS-PAGE analysis.

### SDS-PAGE and immunoblotting.

Equal volumes of protein were mixed with 2× Laemmli sample buffer (Bio-Rad) and boiled at 95°C for 10 min prior to loading onto SDS-PAGE gels (Bio-Rad). Protein was transferred to polyvinylidene difluoride (PVDF) membranes and blocked overnight with 3% bovine serum albumin (BSA) (Sigma) at 4°C. Individual membranes were probed with primary anti-Sca2 or anti-RickA antibody followed by IRDye goat anti-rabbit IgG 800 (LI-COR) secondary antibody (7, 11). Membranes were then probed with mouse β-actin primary antibody (Sigma), followed by IRDye donkey anti-mouse IgG 680 (LI-COR) secondary antibody to assess the protein-loading control. Blotted membranes were visualized using a LI-COR Odyssey CLx. Images were imported into Fiji and analyzed by identifying protein signal values of Sca2 or RickA normalized to respective loading controls.

### Amblyomma maculatum.

Rickettsia-free A. maculatum was either maintained at the Louisiana State University School of Veterinary Medicine (LSU-SVM) using methods previously described or provided by the Centers for Disease Control and Prevention for distribution by BEI Resources, NIAID, NIH (adult A. maculatum, NR-44382) (38–40). All ticks were maintained in a controlled environmental chamber at 27°C with 92% relative humidity and a 12:12 h (light:dark) cycle prior to experimentation.

### *In vivo* A. maculatum dissemination assays via capillary feeding technique.

For dissemination assays, 20 female and 6 to 10 male A. maculatum organisms were encapsulated and prefed on guinea pigs for 3 to 4 days. Subsequently, female ticks were forcibly removed with forceps and restrained for the capillary feeding technique (1, 14). Briefly, ticks were made to adhere to a petri dish dorsum side down, and capillary tubes (25 μl) (Kimble-Chase) containing 2 μl of rhodamine B and Rickettsia, at a concentration of 5 × 10^7^ rickettsiae/μl, were fitted over the hypostome. Ticks were placed in a humidified incubator set at 37°C for 12 h to allow for acquisition of infectious dose. Postincubation ticks were removed from petri dishes and serially washed in 70% ethanol and deionized water to remove nonimbibed media from the tick surface. Ingestion of Rhodamine B was visualized via a fluorescence dissecting microscope. Nonfluorescently labeled A. maculatum was excluded from further study. Positively labeled ticks were separated into three groups: (i) 12 h postexposure (hpe), (ii) 3 days postexposure (dpe), and (iii) 7 dpe. Group i was dissected immediately after capillary feeding. Groups ii and iii were returned to their original host guinea pig and allowed to feed for the designated number of days, at which point they were removed and dissected, and individual midgut, salivary gland, ovaries, and hemolymph tissues were processed for gDNA as previously described (41). A portion of each organ, excluding the hemolymph, was utilized for immunofluorescence assay (IFA) for all ticks and time points. For all tick feeding assays, adult A. maculatum organisms were fed on Hartley guinea pigs (Charles River Laboratories) in accordance with the LSU-SVM Institutional Care and Use Committee (IACUC) under approved protocol number 15-115.

### gDNA extractions and qPCR.

All gDNA was extracted via a Qiagen DNeasy blood and tissue kit (Qiagen) and eluted into 35 μl of RNase/DNase-free water for all *in vitro* and *in vivo* samples. Minor modifications for tick tissue extractions were as follows. Briefly, tick tissues were snap-frozen in liquid nitrogen and ground with a pestle, followed by incubation with proteinase K for 5 h at 56°C. All further steps were carried out according to the manufacturer's protocol. An environmental control extraction was included alongside experimental samples. All samples were evaluated via quantitative real-time PCR (qPCR) on a Roche LightCycler 480II (Roche) using gene-specific primers and probes (Table 1). Amplicons for each primer set were incorporated into pCR4-TOPO, and resultant plasmids were serially diluted to serve as internal standards for each assay. Standards of known copy number, as well as environmental and negative (water) controls, were included along with experimental samples in each set of reactions. Rickettsial infection density was calculated as a ratio of rickettsial copy number to host cell copy number as determined by the absolute quantitation result of qPCR. For *in vivo* tick organs, the ratio was then extrapolated to represent 10^6^ cells, equaling the average A. maculatum cell number quantified via qPCR.

**TABLE 1:** List of primers and probes used for qPCR

Primer set or probe^*a*^	Sequence^*b*^ (5′−3′)	Partial gene amplified	Reference or source
RpompB129FJJ	CAAATGTTGCAGTTCCTCTAAATG	R. parkeri ompB	38
RpompB224RJJ	AAAACAAACCGTTAAAACTACCG		
RparompB	FAM-TTTG+A+G+C+A+G+CA-IABkFQ		
AmacMIF.18F	CCAGGGCCTTCTCGATGT	A. maculatum mif	28
AmacMIF.99R	CCATGCGCAATTGCAAACC		
AmacMIF.63	HEX-TGTTCTCCTTTGGACTCAGGCAGC		
Vero b-actin.61F	TGAAGTGTGACGTGGACATCCATA	Vero β-*actin*	29
Vero b-actin.170 R	GGCAGTAATCTCCTTCTGCATCCT		
Vero b-actin.116	TGGCACCACCATGTACCCTGGCATTGCT		
ISE6_calFW	AGCAGGGAACTTTCAAGCTG	*I. scapularis crt*	This paper
ISE6_calREV	AGAAAGGCTCGAACTTGGTG		
ISE6_cal.67	HEX-AGACCTCTGAAGATGCCCGCTTT		

a A plus sign denotes the use of a locked nucleic acid.

b FAM, 6-carboxyfluorescein; IABkFQ, Iowa Black dark quencher; HEX, hexachlorofluorescein.

### Immunofluorescence assay.

All incubations with coverslips from *in vitro* assays or tick tissues from *in vivo* bioassays were completed at room temperature in a humidified chamber. *In vitro* and *in vivo* samples were washed once in PBS and fixed with 4% paraformaldehyde with 4% sucrose in PBS for 15 min. Samples were permeabilized with 0.1% Triton X-100 in PBS for 15 min and then blocked with 3% BSA in PBS for 1 h. Antibodies were diluted in 1% BSA (Sigma-Aldrich) in PBS. For detection of rickettsiae, RC_PFA_ was utilized followed by goat anti-rabbit Alexa Fluor 488 (Molecular Probes) (42). Samples were washed 3 times with PBS to remove unbound antibody. DAPI (4′,6-diamidino-2-phenylindole dihydrochloride) was utilized for nuclear staining. Cytoskeletal structure of host cells was visualized using Phalloidin-X-Alexa Fluor 568 (Molecular Probes) for *in vitro* samples or Phalloidin-X-Alexa Fluor 647 (Molecular Probes) for *in vivo* tick tissues. A negative-control (no primary antibody) sample was stained in tandem with experimental samples. All samples were mounted with Mowiol (43), sealed, and stored at 4°C until imaged.

### Imaging and analysis.

A TCS SPS confocal microscope (Leica) was utilized for analysis of actin polymerization and Rickettsia infection *in vivo* and *in vitro*. For analysis of *in vitro* actin polymerization, 10 random images were taken for each treatment group and time point. All images were then imported into Fiji software and adjusted for brightness and contrast, followed by using the smoothing tool (44). Actin tails were enumerated using the cell counter plugin. The cell-to-cell spread of rickettsiae *in vitro* was determined by capturing 10 random images across each treatment group at 24 hpi. Infected and noninfected nuclei were then counted to yield the average percentage of infected cells. Postanalysis, all images were adjusted for brightness, contrast, and smoothness in Fiji software. For Image S3 in the supplemental material, a z-stack consisting of 20 image slices was compiled in Fiji by first importing all stacks with the Bio-Formats Importer and then compiled using the z-project tool. All images were compiled into figures using Adobe Illustrator.

### Statistical analysis.

Statistical analysis consisted of Kruskal-Wallis test followed by Dunn's test for growth curve analysis, a *t* test for assessment of actin polymerization, and a one-way analysis of variance (ANOVA) with Tukey's *post hoc* test for the cell-to-cell spread assay and all *in vivo* analyses. All statistical analyses were administered using GraphPad Prism software. A *P* value of <0.05 was considered significant for all experiments. Statistical analysis was completed using non-log-transformed data. For graphical representation, data were presented on a log scale, with error bars representing the means ± standard errors of the means (SEM).

## Supplementary Material

Supplemental material
