# Supplementary material for: Role of Sca2 and RickA in the Dissemination of Rickettsia parkeri in Amblyomma maculatum
Source: Infect Immun. 2018 May 22;86(6):e00123-18. doi: 10.1128/IAI.00123-18 (PMC5964526; doi:10.1128/IAI.00123-18)
Supplement: Supplemental material [file supp_86_6_e00123-18__index.html]

Supplemental material 

# Role of Sca2 and RickA in the Dissemination of Rickettsia parkeri in Amblyomma maculatum

## Supplemental material

- Supplemental file 1 -

  Fig. S1. Actin-based motility of wild-type *R. parkeri* in ISE6 cells.

  PDF, 4.2M
- Supplemental file 2 -

  Fig. S2. Representative confocal microscopy images of *A. maculatum* midgut (A), salivary glands (B), and ovary (C) post-rickettsial exposure.

  PDF, 5.3M
- Supplemental file 3 -

  Fig. S3. Z-stack rendering of wild-type *R. parkeri* infecting the midgut of *A. maculatum* at 3 days postexposure.

  PDF, 3.5M
- Supplemental file 4 -

  Fig. S4. Representative no-primary-control images of the *R. parkeri* wild type in multiple tick organs.

  PDF, 7.6M
- Supplemental file 5 -

  Legends for Fig. S1 to S4.

  DOCX, 13K
