# Supplementary figures and images for: Role of Sca2 and RickA in the Dissemination of Rickettsia parkeri in Amblyomma maculatum
Source: Infect Immun. 2018 May 22;86(6):e00123-18. doi: 10.1128/IAI.00123-18 (PMC5964526; doi:10.1128/IAI.00123-18)

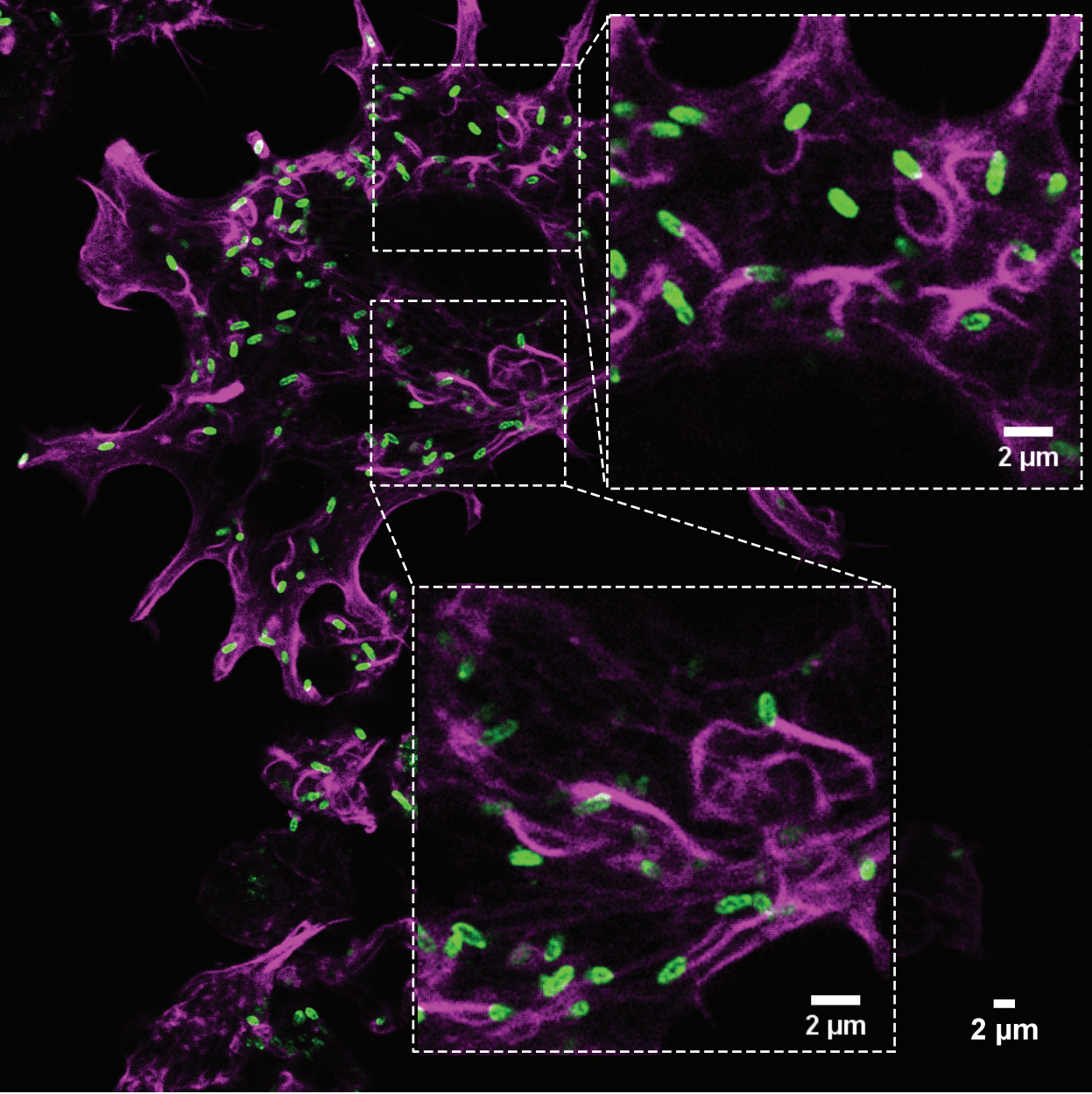

Supplement: Supplemental material [file IAI.00123-18_zii999092418s1.pdf]

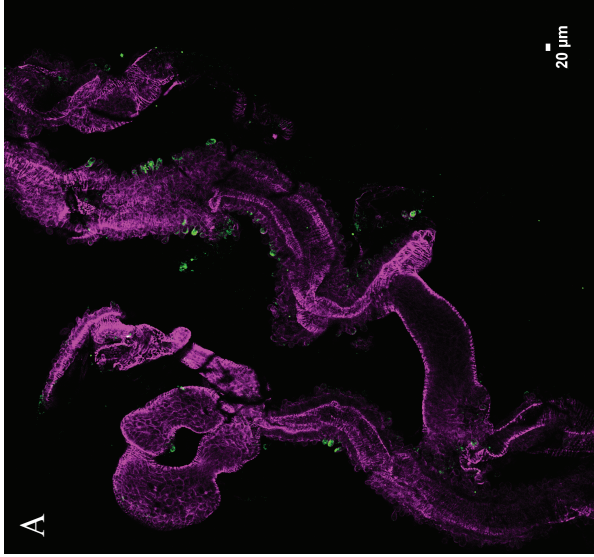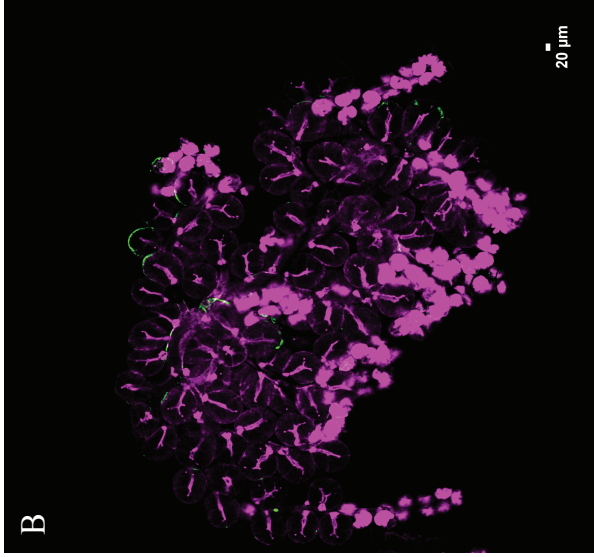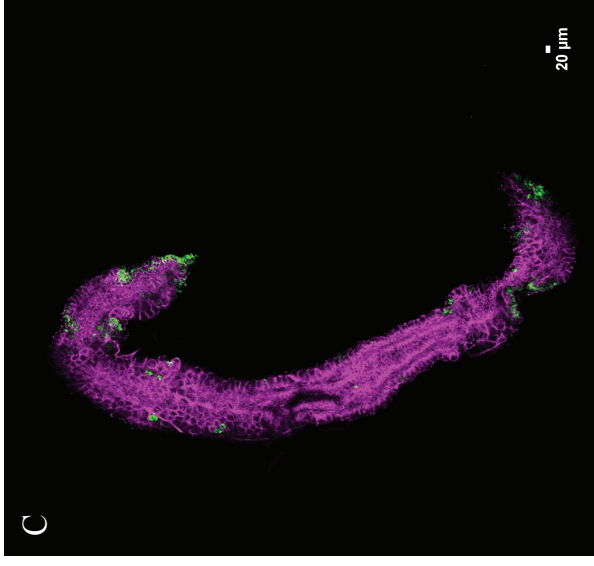

Supplement: Supplemental material [file IAI.00123-18_zii999092418s2.pdf]

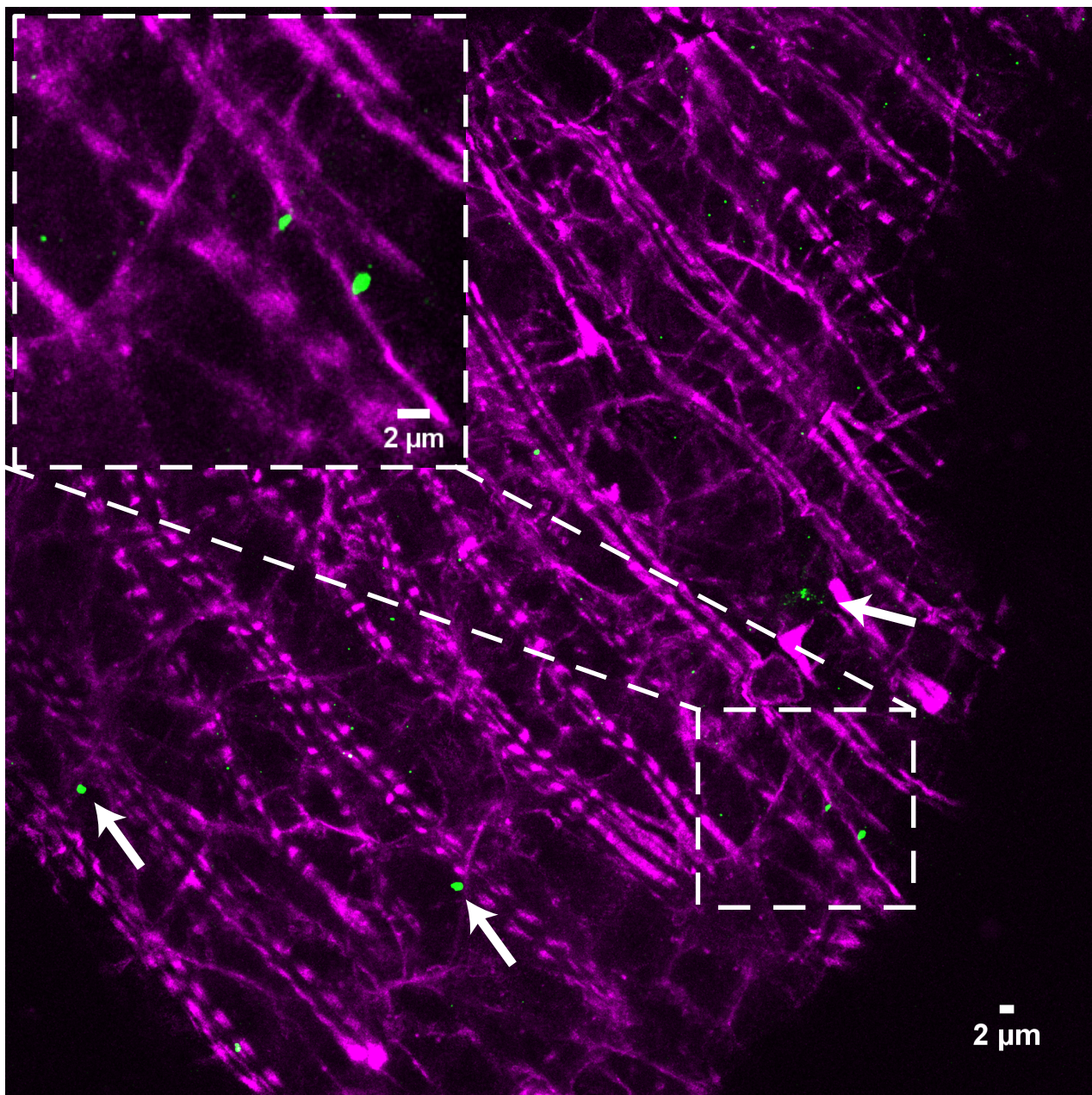

Supplement: Supplemental material [file IAI.00123-18_zii999092418s3.pdf]

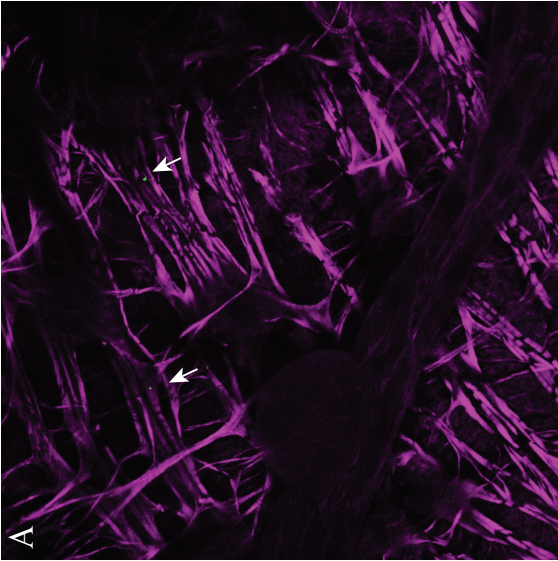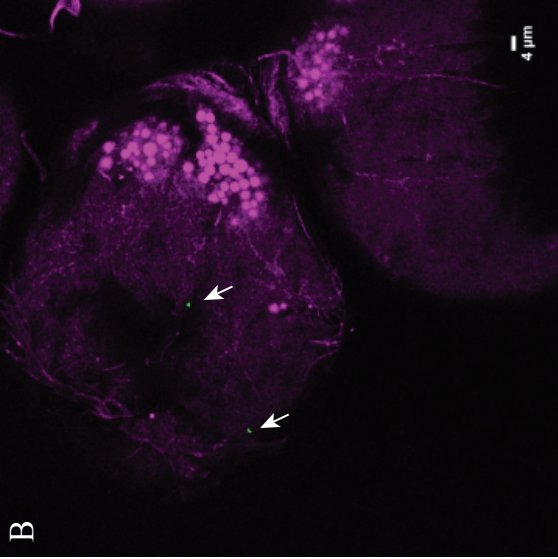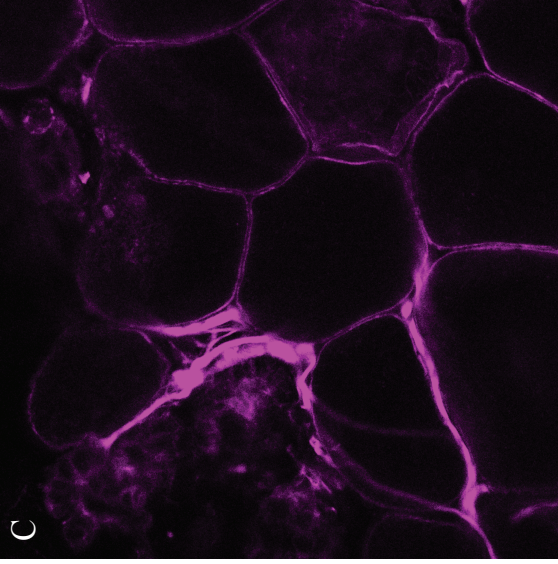

Supplement: Supplemental material [file IAI.00123-18_zii999092418s4.pdf]
