# Supplementary material for: Role of Sca2 and RickA in the Dissemination of Rickettsia parkeri in Amblyomma maculatum
Source: Infect Immun. 2018 May 22;86(6):e00123-18. doi: 10.1128/IAI.00123-18 (PMC5964526; doi:10.1128/IAI.00123-18)
Supplement: Supplemental material [file IAI.00123-18_zii999092418s5.docx]

Supplemental Figure 1. Actin based motility of wild-type *R. parkeri* in ISE6 cells. Inset shows high magnification of rickettsiae with and without actin tails. White scale bar represents 2µm in all images.

Supplemental Figure 2. Representative confocal microscopy images of *A. maculatum* midgut (A), salivary glands (B), and ovary (C) post-rickettsial exposure. All organs were stained for *Rickettsia* (green) and actin (magenta). Scale bar represents 20 μm.

Supplemental figure 3. Z-stack rendering of wild-type *R. parkeri* infecting the midgut of *A. maculatum* at 3 days post-exposure. Inset details high magnification of non-actin polymerizing rickettsiae located in the midgut. White arrows denote rickettsiae. White scale bar represents 2μm.

Supplemental Figure 4. Representative no primary control images of *R. parkeri* wild-type in multiple tick organs. Confocal microscopy of midgut (A), salivary glands (B), and ovaries (C). Cytoskeletal structure of tissues was visualized by staining with actin (magenta). Tissues were incubated with secondary AlexFluor 488 (green) alone to visualize non-specific binding. White scale bar=4μm. Arrows indicate non-specific binding of secondary antibody.
